# The correct use of the ART approach

**DOI:** 10.1590/S1678-77572010000100002

**Published:** 2010

**Authors:** Jo E. FRENCKEN, Soraya Coelho LEAL

**Affiliations:** 1 DDS, MSc, PhD, Associate Professor, Nijmegen International Centre for Oral Health, Radboud University Nijmegen Medical Centre, College of Dental Sciences, Nijmegen, the Netherlands.; 2 MSc, PhD, Associate Professor, Department of Dentistry, School of Health Science, University of Brasília, Brasília, DF, Brazil.

**Keywords:** Atraumatic restorative treatment, Minimal intervention dentistry, Glass-ionomer cements

## Abstract

Confusion exists amongst dentists and scientists about the correct use of the caries management approach termed atraumatic restorative treatment (ART). Inconsistent use of the original definition of ART and suggested modifications (mART) have led to misunderstanding, misconception and miscommunication in the dental literature over the last decade. The aim of this paper is to contribute to a uniform understanding and use of the term ART. Adherence to its original description is suggested and two major aspects were addressed: the use of hand instruments only and the use of adhesive materials and systems.

## MINIMAL INTERVENTION DENTISTRY FOR CARIES MANAGMENT

Minimal intervention dentistry (MID) is based on three aspects: 1) the best understanding of the disease etiology and prognosis; i.e. early disease detection and treatment; 2) prevention by the patient, through education and availability of means enabling him/her to take responsibility for his/her own oral healthcare, and by the dental professional, through application of preventive measures; 3) tissue preservation treatments for cavitated lesions through the use of minimally invasive operative interventions^19,28^.

**Figure 1:**
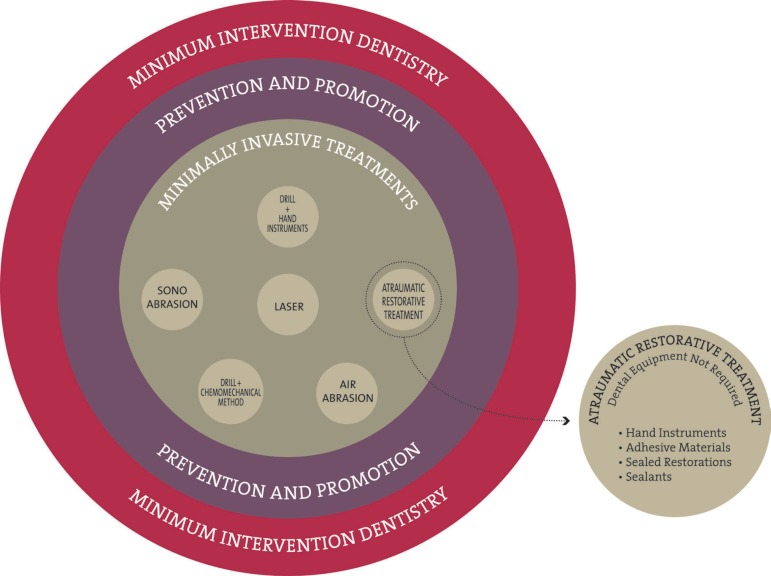
Atraumatic Restorative Treatment within minimum intervention dentistry

Ultraconservative treatment approaches are recommended for treating cavitated dentin lesions^16,28^. These approaches share a common important characteristic: preservation of as much sound tooth structure as possible^24^. However, they also differ; particularly in their implementation phase. For example, different instruments can be used to open and clean cavities^13^. It has been proven that hand instruments can preserve more dental tissue than rotary instruments^1,4^, but hand excavation of carious tissue is a much more time-consuming procedure to be completed^1,4,23,29^. Likewise, using rotary instruments is less time-consuming than using a chemomechanical caries removal gel^1,20^. Therefore, while deciding which approach is most appropriated for a patient, it is of paramount importance that the dentists know the treatment options and are familiar with their advantages and limitations. In order to avoid misinterpretation, they should be aware of requirements involved in performing each of the MID approaches, as the differences between them are subtle (Figure 1).

## THE ATRAUMATIC RESTORATIVE TREATMENT (ART)

ART is one example of the MID concept^8^. It consists of two components: sealing of caries prone pits and fissures with a sealant, and use of a sealant in combination with restoring cavitated dentin lesions^6,9^. The main difference between the ART approach and other minimally invasive operative interventions is that ART uses hand instruments only. Thus, when ART is used either to seal pits and fissures or to restore tooth cavities, hand instruments are used in conjunction with adhesive materials or systems^6,9^. However, in practice, glass-ionomer cement (GIC) has become the most predominantly used material mainly because of its delayed setting reaction that allows handling of the material before it is completely set. Composite resin has also been used to restore primary molars with hand instruments only^5,27^. Polymerization of the material by the use of cord or cordless curing devices is considered as part of the ART approach.

It has been advocated that infection control is simplified when hand instruments for cavity cleaning are used because they can readily be cleaned and sterilized^3^. However, this does not imply that providing ART is simple. Placing ART sealants and ART restorations requires detailed diagnosis, careful observation of the dental structures, and correct and careful performance of all the technical steps in order to produce long-lasting sealants and restorations^17^. According to Bresciani^2^, simplicity of an action does not imply that it should be carried out in a neglectful way. Therefore, attending sufficiently long training sessions is essential to produce successful ART sealants and ART restorations^9,15,28^. Anecdotal information has considered partial excavation of infected dentine being part of the ART approach^25^. Similarly, indirect pulp capping has been ascribed as an ART procedure^11^. It is realistic to expect inexperienced or inadequately trained operators to perform ART restorations less well than trained ones. This has been shown by an operator effect reported in numerous studies^6,7,9,23^.

A number of aspects of the ART approach have been investigated extensively and outcomes have shown that it can be considered an economical and effective method for preventing and controlling carious lesion development in vulnerable populations^21^. It also causes less discomfort and less dental anxiety than the traditional approach using rotary instruments in both adult and pediatric patients^10,18,26^. However, it is accepted that ART cannot be used in all clinical cases and that other treatment methods, mostly those using rotary instruments, are then required. In line with conventional concepts in Cariology and restorative dentistry, we consider the use of rotary instruments followed by cleaning of the cavity with excavators and restoration with an adhesive material to be the normal conventional management of cavitated dentin lesions. This approach is propagated as part of MID^12,13^.

Louw, et al.^13^ studied the ART approach in comparison to that of minimal intervention treatment (MIT) in primary dentitions. In their study, the difference between ART and MIT technique rested on the fact that in ART, cavity opening had to be large enough or could be widened sufficiently with hand instruments to allow the smallest excavator to enter. The MIT used burs mounted in a low-speed handpiece to gain access to the cavity. This is a good example that demonstrates that the use of burs for opening the cavity refers to a different caries management approach than ART. Nevertheless, some researchers have argued that the use of rotary instruments to open the cavity is just an adaptation to the original ART technique proposed 20 years ago^9^. However, to which extent does such an alteration interfere with the ART rationale?

## WHAT IS UNDERSTOOD BY 'MODIFIED ART'?

The term 'modified ART' appears frequently in the dental literature^13^. A modification to the original ART might refer to the fact that the ART approach has been carried out in places where traditional dental equipment has been available instead of in field situations^10^. However, modification is most often associated with the use of rotary equipment: the drill, to open the tooth cavity, followed by the normal ART procedure in cleaning and restoring the cavity. It has been suggested that the use of rotary equipment would make the total procedure quicker and easier^3^. Mainly for those inadequately trained in pure ART, this may be true. However, is the use of rotary instruments really faster? Although it has been reported^5^ that ART using hand instruments is more time consuming when compared to ART using rotary instruments, the literature does not have enough and consistent information concerning this aspect, indicating that further investigations are required. Nevertheless, time is only a minor aspect of the total caries management process and might not be the most important one. More important factors are the smaller cavities resulting from preparation with hand instruments, preserving tooth structures, the reduced pain and the good results concerning survival of ART restorations^14,22,23^.

As the ART approach is increasingly used in a growing number of developing and developed countries, it needs to be ensured that communication amongst its users and researchers can be carried out without misconceptions. The most important requisite for achieving this is the use of the original description of the ART approach, explained in a previous publication^6^ and in a recently released textbook of Cariology^9^. It reads as follows: 'ART is a minimally invasive approach to both prevent dental caries and to stop its further progression. It consists of two components: sealing caries prone pits and fissures and restoring cavitated dentin lesions with sealant-restorations. The placement of an ART sealant involves the application of a high-viscosity glass-ionomer that is pushed into the pits and fissures under finger pressure. An ART restoration involves the removal of soft, completely demineralised carious tooth tissue with hand instruments. This is followed by restoration of the cavity with an adhesive dental material that simultaneously seals any remaining pits and fissures that remain at risk'^9^.

The implication is that no mention should be made of modified ART, as that approach refers to the current conventional concept of treating cavitated lesions^12^.

## CONCLUSION

The Atraumatic Restorative Treatment (ART) is an example of the contemporary caries management philosophy of minimal intervention dentistry. In its principle, it differs from other examples of minimally invasive treatments. This suggests that the term 'ART' should be used in future communication in accordance with its original description.
